# Willingness to accept H1N1 pandemic influenza vaccine: A cross-sectional study of Hong Kong community nurses

**DOI:** 10.1186/1471-2334-10-316

**Published:** 2010-10-29

**Authors:** Samuel YS Wong, Eliza LY Wong, Josette Chor, Kenny Kung, Paul KS Chan, Carmen Wong, Sian M Griffiths

**Affiliations:** 1 School of Public Health & Primary Care, The Chinese University of Hong Kong, Hong Kong, PR China; 2 Department of Family Medicine, Prince of Wales Hospital, Hospital Authority, Hong Kong, PR China; 3 Department of Microbiology, Faculty of Medicine, The Chinese University of Hong Kong, Hong Kong, PR China

## Abstract

**Background:**

The 2009 pandemic of influenza A (H1N1) infection has alerted many governments to make preparedness plan to control the spread of influenza A (H1N1) infection. Vaccination for influenza is one of the most important primary preventative measures to reduce the disease burden. Our study aims to assess the willingness of nurses who work for the community nursing service (CNS) in Hong Kong on their acceptance of influenza A (H1N1) influenza vaccination.

**Methods:**

401 questionnaires were posted from June 24, 2009 to June 30, 2009 to community nurses with 67% response rate. Results of the 267 respondents on their willingness to accept influenza A (H1N1) vaccine were analyzed.

**Results:**

Twenty-seven percent of respondents were willing to accept influenza vaccination if vaccines were available. Having been vaccinated for seasonable influenza in the previous 12 months were significantly independently associated with their willingness to accept influenza A (H1N1) vaccination (OR = 4.03; 95% CI: 2.03-7.98).

**Conclusions:**

Similar to previous findings conducted in hospital healthcare workers and nurses, we confirmed that the willingness of community nurses to accept influenza A (H1N1) vaccination is low. Future studies that evaluate interventions to address nurses' specific concerns or interventions that aim to raise the awareness among nurses on the importance of influenza A (H1N1) vaccination to protect vulnerable patient populations is needed.

## Background

The 2009 pandemic of influenza A (H1N1) infection has alerted many governments to make preparedness plan to control the spread of influenza A (H1N1) infection. With evidence on the effectiveness of vaccination in the control and prevention of seasonal influenza [1,2], vaccination for pandemic influenza is one of the most important primary preventative measures to reduce the disease burden associated with influenza A (H1N1) infection [3].

Several high risk groups have been identified as "the priority group" to receive the influenza A (H1N1) vaccination and among these, healthcare workers have been identified "as a first priority" to be vaccinated against influenza A (H1N1) by the World Health Organization [4,5]. Although it is considered essential for all healthcare workers to be immunized against influenza A (H1N1) to prevent the spread of influenza A (H1N1) to patients as the pandemic evolves, previous studies that have examined the acceptability of seasonal influenza vaccination among healthcare workers have generally demonstrated a low acceptance rate of vaccination in this group [6,7]. Among all healthcare workers, nurses constitute the largest group with the highest frequency of contacts with patients and staff [8]. Previous findings of the acceptability of seasonal influenza vaccination in nurses showed that their acceptance of vaccination was lowest among all healthcare workers [6,7,9,10]. Acceptability of influenza A (H1N1) vaccination in healthcare workers has been shown to be low [11-13]. A survey conducted in Greece found that only 17% of hospital healthcare were willing to receive influenza A (H1N1) vaccination [11]. Of all healthcare workers, nurses were found to have the lowest rate of acceptability of influenza A (H1N1) vaccination [12,13]. A study of Italian healthcare workers showed 31% of nurses willing to accept vaccination compared to 67% of physicians [12]. In a study conducted of Hong Kong healthcare workers in hospitals, it was found that only 25% of nurses were willing to accept influenza A (H1N1) vaccination, compared with 47% of doctors and 29% of allied professionals [13]. General practitioners working in the community in France also report a high rate of acceptability of influenza A (H1N1) vaccination at 62% [14]. It is therefore not surprising that a recent online poll conducted in the UK suggested that nurses may be unwilling to receive pandemic influenza vaccination [15]. In a cross-sectional survey that was conducted on experienced nurses who were members of the nursing professional organizations in Hong Kong, the vaccination rate for seasonal influenza vaccination was about 50% [16]. In a more recent survey that explored influenza A (H1N1) acceptance rate in the same group of nurses [17], it was found that only 13% were willing to accept vaccination for influenza A (H1N1) compared to 38% who plan to receive the seasonal influenza vaccination. However, in the study, there was a low response rate of 28% of nurses with different clinical settings. There is a lack of studies in Hong Kong looking at influenza A (H1N1) vaccination acceptability particularly in the community setting. Nurses who work in the community may be the first group to be in contact with patients who are affected with the influenza A(H1N1) infection. A recent study [18] showed differences in the concerns in using new vaccines during a pandemic than using established vaccine in a non-crisis situation. Therefore, we undertook the current study to examine the willingness of frontline registered nurses who work in the community in Hong Kong to receive vaccination against influenza A (H1N1) at the time of a pandemic.

## Methods

### Participants

All participants in this study were specially trained nurses, who provided nursing care and treatment for patients in their own homes (also known as Community Nursing Service) in Hong Kong. The responsibility of these community nurses is to provide nursing care and health education to patients through home visits. CNS nurses are employed by Hospital Authority in Hong Kong and provide continuity of care for patients who have been discharged from hospitals such that patients can recover in their own homes. Community nurses were chosen because of their frequent contacts with patients in their homes which is likely to increase their risk for exposure to influenza. We have only included CNS nurses who provide medical services in the study. The rest of the CNS nurses (around 100 nurses) provide psychiatric services in the community.

Currently, there are a total of 401 nurses who provide medical related services for the Community Nursing Service (CNS) centres that are distributed among the 7 geographical clusters in Hong Kong (in Hong Kong, public hospital and primary care services are organized in 7 clusters that covers all of Hong Kong).

In this study, twelve major CNS centres were contacted first and all CNS nurses were invited to participate in the current study through these 12 major centres. All 12 centres responded to this study and 270 questionnaires were returned with 267 completed questionnaires [19]. The response rate for this study was 67% and all questionnaires were received within a 2-week period at a time when there was widespread H1N1 in the community.

### Study Design

The survey was sent out from June 24^th ^to June 30^th^, 2009 when the WHO influenza pandemic alert level assigned to H1N1 was phase 6. Phase 6 signifies a widespread human infection, indicating that the virus has caused sustained community level outbreaks in at least one other country in another WHO region (WHO pandemic phase description). The pandemic in Hong Kong started on 1^st ^May, when a Mexican traveller was confirmed with influenza A (H1N1). Till the end of our data collection, there were 1389 confirmed cases and no death were reported.

All general managers of the involved community nursing centres were contacted through telephone to obtain approval to send questionnaires to their nursing staff. In total, 401 self administered, anonymous questionnaires were posted to general managers of centres who then passed these questionnaires to the community nurses in their centres. The general managers of centres were then reminded via telephone during the period from 2^nd ^July and 8^th ^July one week after the questionnaires were sent out and advised to return the completed questionnaires within the week. Once completed, questionnaires were collected and returned by their supervisors, except for one of the (Sau Mau Ping) sub-offices, where nurses mailed back their questionnaires individually. All centres sent their questionnaires back after one telephone reminder. The last pile of completed questionnaires was received on 14^th ^July, 2009.

### Survey design

The questionnaire consisted of six parts with 44 questions and the full questionnaire can be accessed by contacting the authors. The first four parts were based on a conceptual framework developed by Patel et al [20] to guide systematic planning for community primary care service response to pandemic influenza with modifications to make it more relevant for nurses. We added a fifth part on psychological responses to pandemic influenza and a sixth part on demographics of respondents which were based on two studies previously published (one on general practitioners' response to SARS and one on general public response to swine flu) [21,22]. In summary, these sections were 1) clinical services change as a response to pandemic influenza; 2) internal environment changes as a response to pandemic influenza e.g. wearing of mask; 3) macro-environmental changes as a response to pandemic influenza e.g. use of guideline etc; 4) professional and public health responsibilities with respect to pandemic influenza; 5) attitude and psychological responses to pandemic influenza; and 6) demographics and year of education of respondents. The willingness to accept influenza A(H1N1) vaccination was asked in the professional and public health responsibility sections and the question "will you receive the new influenza A (H1N1) vaccine when it is available" was asked with a dichotomous "yes" or "no" response. For those who answered no, they were further asked to give their reasons for refusing to receive the vaccine. Only results on willingness of accept influenza A (H1N1) vaccination and information related to the analysis on willingness to accept vaccine are reported in this paper. Other results from this survey will be presented in a separate report.

### Statistical analysis

Descriptive results were cross-tabulated. χ^2 ^test was used to examine characteristics between nurses who were willing to accept influenza A (H1N1) vaccination against those who were not willing to accept vaccine. Univariate analysis was performed with demographic information (age, post year education and working district), personal protective behaviour (hand washing practice), experience of taking care of SARS patients, and influenza vaccination in the previous 12 months as independent variables. Dependent variables were the willingness to receive pandemic influenza vaccination. Multiple logistic regression analysis was conducted to examine the relationship between pre-defined factors that we think might be associated with the acceptance of the influenza A (H1N1) vaccine when constructing the questionnaire and the dependent variable. The level of statistical significance was set at a p-value of ≤ 0.05.

## Results

### Demographics

Among the respondents (Table 1), most of them were females who had worked an average of 8.8 years as a community nurse (ranging from 2 months to 32 years) and having been a registered nurse for 16.5 years (ranging from 1 year to 36 years). The mean age of respondents was 39.1 years and about a third (30%) had had the experience of dealing with SARS. One third of them had received vaccination for seasonal influenza in the past 12 months. Nurses from each geographical cluster in Hong Kong participated, with 11% of respondents working in Hong Kong Island, 47% working in Kowloon and 42% working in the New Territories (Hong Kong is geographically divided into Hong Kong Island, Kowloon peninsula and the New Territories).

**Table 1:** Characteristics on nurses' willingness to receive influenza A (H1N1) vaccination

	Total Number	Number of nurses declining	Number of nurses accepting
**Age (n = 224)**			
< = 39 years	111 (50%)	82 (74%)	29 (26%)
39 years +	113 (50%)	83 (74%)	30 (26%)

**Gender (n = 259)**			
Female	249 (96%)	185 (74%)	64 (26%)
Male	10 (4%)	6 (60%)	4 (40%)

**District (n = 253)**			
Hong Kong Island	28 (11%)	24 (86%)	4 (14%)
Kowloon	119 (47%)	88 (74%)	31 (26%)
New Territories West	63 (25%)	47 (75%)	16 (25%)
New Territories East	43 (17%)	28 (65%)	15 (35%)

**Postgraduate (n = 255)**			
No	61 (24%)	45 (74%)	16 (26%)
Yes	194 (76%)	142 (73%)	52 (27%)

**SARS or Avian Flu experience (n = 262)**			
No	184 (70%)	137 (75%)	47 (25%)
Yes	78 (30%)	57 (73%)	21 (27%)

Received vaccination in the past 12 months* (n = 261)			
No	172 (66%)	139 (81%)	33 (19%)
Yes	89 (34%)	54 (61%)	35 (39%)

**Wash hands after taking patients in outreach service (n = 252)**			
No	49 (19%)	33 (67%)	16 (33%)
Yes	203 (81%)	154 (76%)	49 (24%)

*p < 0.001

### Willingness to accept vaccination and characteristics

Overall, 194 (73%) participants do not want to receive new influenza A (H1N1) vaccine when it is available. The reasons for their not intending to receive vaccination when it is available are summarised in Table 2.

**Table 2:** Reasons for declining influenza A (H1N1) vaccine

Reasons	N (%) of nurses declining
Effectiveness	105 (59)
Side-effects	149 (83)
Production Site	49 (27)
Other concerns (i.e. pregnancy, poor health status, and the severity of the epidemic of H1N1)	10 (<0.1)

Note: The total percentage exceeds 100% because multiple responses were allowed.

The characteristics of respondents who were willing to accept influenza A (H1N1) vaccination and with those who were not willing to accept influenza A (H1N1) influenza vaccination were compared by χ2 test and were presented Table 1. Nurses who were willing to receive influenza A (H1N1) vaccine were different from nurses who were not willing to receive influenza A (H1N1) vaccine with respect to "being vaccinated against seasonal influenza vaccination in the previous 12 months". There were no statistical significant differences in other characteristics as analyzed by chi-square test.

The relationship between demographic and other characteristics of the nursing respondents and their willingness to accept vaccination were analyzed further using forced entry logistic regressions (Table 3). Having seasonal vaccination in the past 12 months was significantly independently associated with the willingness to accept influenza A (H1N1) vaccination (OR = 4.03; 95% IC: 2.03-7.98). Washing hands before and between patient contact, however, was negatively independently associated with willingness to accept influenza A (H1N1) vaccination (OR = 0.49; 95% IC: 0.23-1.06). To confirm the results, we have also conducted backward logistic regression and the results also indicated that having seasonal vaccination in the past 12 months was significantly associated with the willingness to accept influenza A (H1N1) vaccination (OR = 3.56, 95% CI: 1.87-6.80, p < 0.001).

**Table 3:** Independent association between demographics, nurses' behaviours experiences and their willingness to receive influenza A (H1N1) vaccination

Variables and variable levels	β	Odds ratio	95% CI	*p *value
**Age**				
≤39 years		Reference		
>39 years	0.28	1.32	0.67 - 2.61	0.418

**District**				
Hong Kong Island		Reference		
Kowloon	0.56	1.74	0.45 - 6.73	0.421
New Territories West	0.83	2.28	0.55 - 9.45	0.255
New Territories East	1.46	4.31	0.98 - 18.93	0.053

**Postgraduate**				
No		Reference		
Yes	-0.51	0.60	0.27 - 1.33	0.207

**SARS or Avian Flu experience**				
No		Reference		
Yes	-0.27	0.76	0.36 - 1.62	0.476

**Received vaccination in the past 12 months**				
No		Reference		
Yes	1.39	4.03	2.03 - 7.98	<0.001

**Wash hands in outreach service**				
No		Reference		
Yes	-0.71	0.49	0.23 - 1.06	0.068

## Discussion

Consistent with findings from previous surveys conducted in hospital healthcare workers and nurses [13,17], we have shown that the majority of nurses from community nursing services in Hong Kong were not willing to be vaccinated against H1N1 influenza when the vaccine becomes available. Similar to findings from previous studies in healthcare workers [13,17,23,24], we showed that the major concerns for vaccination against pandemic influenza was fear of side effects and concern of efficacy of the new vaccine (Table 2). Moreover, influenza vaccination in the previous 12 months was significantly associated with their willingness to accept the pandemic influenza vaccination.

We also showed that in addition to previous vaccination with seasonal influenza, preventive behaviours such as frequent hand washing practice were independently associated with nurses' willingness to accept influenza A (H1N1) vaccination. We showed that "have been washing hands between and before patient contact" was negatively associated with willingness to accept vaccination independently although the reason for this is unclear and be a result of our relatively small sample. We can only postulate that the barrier to pandemic influenza vaccination is probably not related to the willingness of nurses to protect themselves against infections or their personal hygiene in general. Researchers [17] have suggested one of the barriers to pandemic influenza vaccination in nurses was misconceptions about the purpose of vaccinations in which nurse might think that the aim of vaccination was for self protection rather than to protect at risk populations in contact with them [17,23,25]. Specific vaccination policy for health care workers may improve vaccination in this group as nurses have different concerns and priorities when compared to the general public's concerns [17,25].

Although some may suggest that more educational programs for healthcare workers may be a solution to the low vaccination uptake [13], studies have reported low influenza vaccination rates among healthcare workers even when educational programs were implemented [10]. Other studies including randomized controlled trials also failed to show that better knowledge or educational programmes were effective in increasing acceptability of vaccination in healthcare workers [26]. Indeed, some suggested that educational campaigns based on the Health Belief Model were unlikely to be enough to change healthcare workers' acceptability of vaccination as evidence showed that perceived seriousness of infection, acknowledgement of increased risk of infection and knowledge of vaccine being safe were unrelated to vaccine uptake in healthcare workers [26]. Others suggested that educational programmes may be counter-productive as many of these healthcare workers do not perceive themselves to be at risk for contracting the infection. Recently, Ofstead et al [25] suggested that an ecological model, which included engaging organizations, communities and policy makers to create environments that were more conducive to risk reduction, might be more effective in increasing vaccination rates in healthcare workers.

### Strengths and weaknesses of the study

To our knowledge, this is the first study to explore the willingness of nurses who work in the community to be vaccinated for pandemic influenza and our results confirmed that their acceptability of influenza A (H1N1) vaccination is low. A strength of our study is our response rate of 67% which is higher than similar report conducted in Hong Kong with a response rate of 28% [18].

A limitation of our study is that we have only documented nurses' intentions of when a vaccine is available and not the actual uptake of vaccination. Furthermore, all data from this study were from self-reports and recall bias, such recalling influenza vaccination in the previous year, might have occurred. A possible contributory factor e.g. recent episode of influenza-like illness which may influence the willingness of vaccination was not enquired. Our analysis of results was limited by the relatively small sample size in nurses who are part of the Community Nursing Service in Hong Kong with no information available on non respondents. However, our results are similar to recent studies conducted in hospital healthcare workers [13] and members of professional nursing organizations [17] in Hong Kong.

## Conclusions

Consistent with previous findings which were conducted in healthcare workers and nurses [13,17], we confirm that the acceptance rate of pandemic influenza vaccination is low amongst community nurses. Since community nurses are at high risk of contracting influenza infection, and play a significant role in caring for community cases, special attention should be paid to this group as successful vaccination strategy has been shown to be beneficial in disease transmission [27]. Future work, including interventional studies evaluating potential interventions based on the ecological model or interventions that aim to increase awareness among nurses on the importance of vaccination in healthcare workers to protect vulnerable populations [16] is needed. The need to address low influenza vaccination rates in this high-risk group is urgent in the context of pandemic response.

## Competing interests

The authors declare that they have no competing interests.

## Authors' contributions

All authors were involved in the design of the project. The survey tool was designed by SYSW and ELYW. The data collection, analysis and the results interpretation were carried by SYSW and ELYW in consultation with the team. The first draft of this article was composed by SYSW and was revised critically by all authors. All authors have approved the final version of the manuscript.

## Pre-publication history

The pre-publication history for this paper can be accessed here:

http://www.biomedcentral.com/1471-2334/10/316/prepub
